# Long-term epilepsy-associated tumors: transcriptional signatures reflect clinical course

**DOI:** 10.1038/s41598-019-56146-y

**Published:** 2020-01-09

**Authors:** Daniel Delev, Karam Daka, Sabrina Heynckes, Annette Gaebelein, Pamela Franco, Dietmar Pfeifer, Marco Prinz, Oliver Schnell, Horst Urbach, Irina Mader, Jürgen Beck, Alexander Grote, Albert J. Becker, Dieter Henrik Heiland

**Affiliations:** 1 Department of Neurosurgery, Medical Center, Freiburg, Germany; 2 Department of Neurosurgery, RWTH University Aachen, Aachen, Germany; 3 Translational NeuroOncology Research Group, Medical Center, University of Freiburg, Freiburg, Germany; 4 Department of Hematology, Oncology and Stem Cell Transplantation, Medical Center University of Freiburg, Freiburg, Germany; 5 Signalling Research Centres BIOSS and CIBSS, University of Freiburg, Freiburg, Germany; 6 Center for NeuroModulation (NeuroModul), University of Freiburg, Freiburg, Germany; 7 Institute of Neuropathology, Medical Center, University of Freiburg, Freiburg, Germany; 8 Department of Neuroradiology, Medical Center, University of Freiburg, Freiburg, Germany; 9 Clinic for Neuropediatrics and Neurorehabilitation, Epilepsy Center for Children and Adolescents, Schön Klinik, Vogtareuth, Germany; 10 Clinic for Neurosurgery, Evangelic Hospital of Bethel, Bielefeld, Germany; 11 Section for Translational Epilepsy Research, Department of Neuropathology, University of Bonn Medical Center, Bonn, Germany; 12 Medical Faculty, Freiburg University, Freiburg, Germany

**Keywords:** Epilepsy, CNS cancer

## Abstract

Long-term epilepsy-associated tumors (LEATs) represent mostly benign brain tumors associated with drug-resistant epilepsy. The aim of the study was to investigate the specific transcriptional signatures of those tumors and characterize their underlying oncogenic drivers. A cluster analysis of 65 transcriptome profiles from three independent datasets resulted in four distinct transcriptional subgroups. The first subgroup revealed transcriptional activation of STAT3 and TGF-signaling pathways and contained predominantly dysembryoplastic neuroepithelial tumors (DNTs). The second subgroup was characterized by alterations in the MAPK-pathway and up-stream cascades including FGFR and EGFR-mediated signaling. This tumor cluster exclusively contained neoplasms with somatic *BRAF*^*V600E*^ mutations and abundance of gangliogliomas (GGs) with a significantly higher recurrence rate (42%). This finding was validated by examining recurrent tumors from the local database exhibiting *BRAF*^*V600E*^ in 90% of the cases. The third cluster included younger patients with neuropathologically diagnosed GGs and abundance of the NOTCH- and mTOR-signaling pathways. The transcript signature of the fourth cluster (including both DNTs and GGs) was related to impaired neural function. Our analysis suggests distinct oncological pathomechanisms in long-term epilepsy-associated tumors. Transcriptional activation of MAPK-pathway and *BRAF*^*V600E*^ mutation are associated with an increased risk for tumor recurrence and malignant progression, therefore the treatment of these tumors should integrate both epileptological and oncological aspects.

## Introduction

Tumor series derived from epilepsy-surgery programs cover a range of generally rare glial and glioneuronal entities. Among them, gangliogliomas (GGs) and dysembryoplastic neuroepithelial tumors (DNTs) represent the most common glioneuronal tumors causing tumor-related drug-resistant epilepsy in young adults^1^. Since epilepsy is the leading clinical symptom of these tumors, GGs and DNTs (together with pleomorphic xanthoastrocytomas, PXAs) are often referred to as long-term epilepsy-associated tumors (LEATs)^2,3^. While for common glial entities such as pilocytic astrocytomas (PAs) and PXAs the diagnostic criteria and parameters determining the clinical outcome are well established^4^, the situation is more difficult for glioneuronal tumors. For GGs and DNTs, the neuropathological stratification is particularly complex with high inter-observer variability^5^. This is resembled by inconsistent diagnose frequencies, showing variations form 6% to 49% for GGs and 7% to 80% for DNTs in large series^6^. Striving for more reliable diagnostic approaches, recent studies started investigating the molecular background of LEATs showing a significant presence of genetic alterations involving the MAPK and mTOR pathways^7–12^. For the first time, a recent study by Stone *et al*. presented a molecular characterization of epilepsy-associated glioneuronal tumors based on both methylation and expression profiles, identifying two distinct molecular groups, which corresponded only partially to their histopathological appearance^13^.

LEATs are regularly treated by surgical resection with seizure free rates ranging from 77% to 93% for DNTs and 63% to 100% for GGs^14,15^. Although seizure relief is of a paramount importance, it is the oncological aspect, which distinguishes indications and surgical strategy in patients with glioneuronal tumors from other “epilepsy-typical” resections including focal cortical dysplasias (FCDs) and hippocampal sclerosis. LEATs are indeed predominantly benign tumors, but a small subgroup of GGs and DNTs show tumor progression and even malignant transformation, implying that the histopathological appearance may not represent entirely the underlying tumor biology^16–20^. Consequently, the oncological aspect should be also considered during the treatment decision-making process^21^.

Here, we present a comprehensive clinical, pathological and molecular analysis aiming at the more precise differentiation of long-term epilepsy-associated tumors and better understanding of their oncological background. We performed transcriptional analysis identifying four distinct molecular subgroups among histologically diagnosed GGs, DNTs. and PXAs. Gene Set Enrichment Analysis (GSEA) identified genetic abnormalities characteristic for each subgroup helping to stratify those patients, who carry a higher risk for tumor progression. Finally, the transcriptional results were validated by immunohistochemistry examinations of recurrent epilepsy-associated glioneuronal tumors from the local patient database.

## Results

### Clustering revealed distinct transcriptional architecture of long-term epilepsy associated tumors

**Figure 1:**
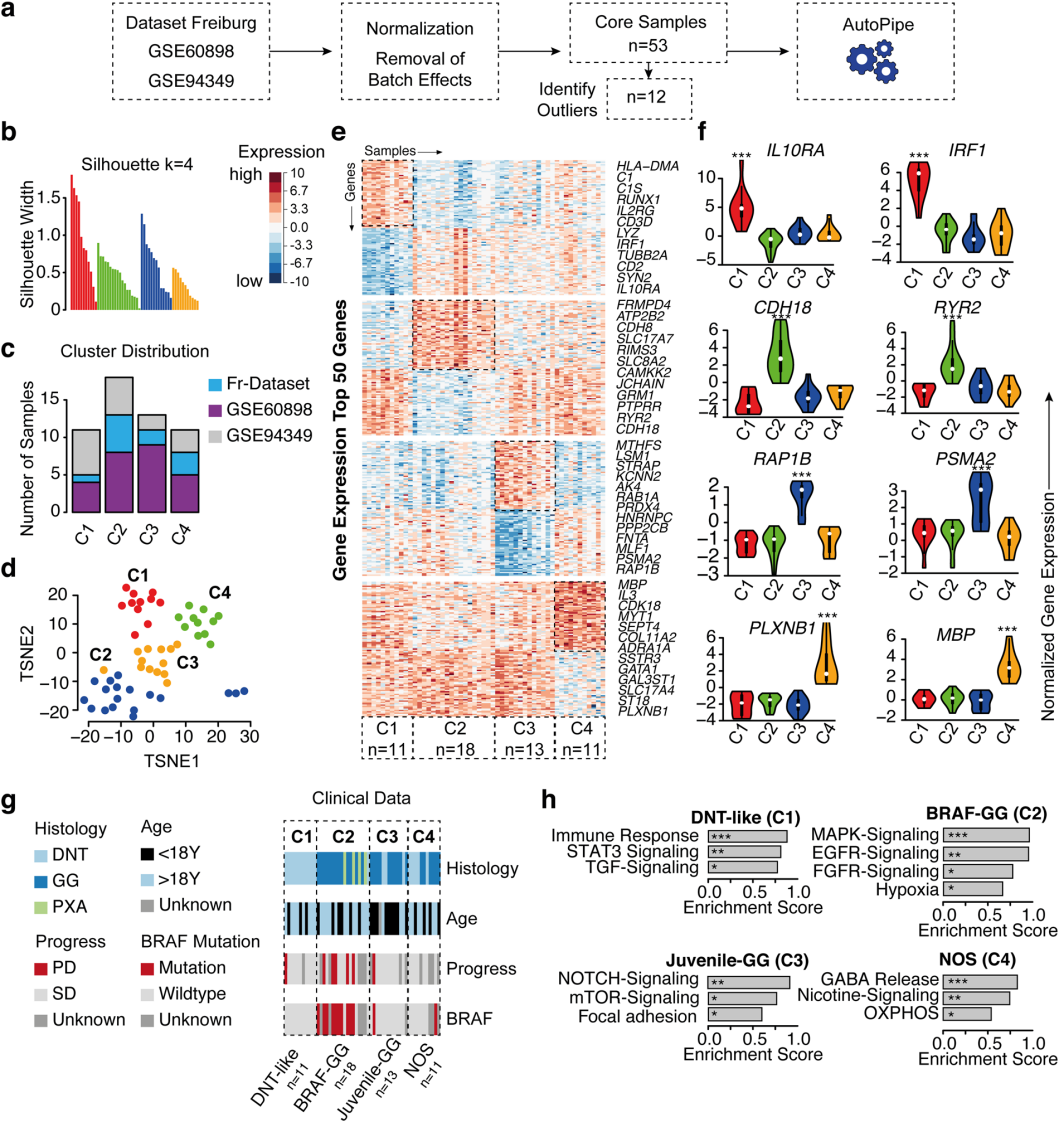
Transcriptional signature of long-term epilepsy-associated tumors. (**a**) Illustration of the workflow. Dataset collection including our own cohort and 2 GEO databases, which were integrated into our analysis (GSE60898 and GSE94349). (**b**) Bar-plot of the silhouette widths of each patient based on the “PAM” cluster integrated in the AutoPipe-package (CRAN). The optimal number of clusters was computed by “PAM” clustering and visualized by the mean silhouette widths. Patients with negative silhouette widths were excluded. Full cluster are given in the Supplementary Fig. 1. (**c**) Sample distribution among all integrated datasets (**d**) tSNE visualization of the cohort. Colors indicate the cluster origin. (**e**) A heatmap with distinct up- and downregulated genes of each cluster group (C1–C4). *Red* indicates up-regulated gene expression, *blue* down-regulated genes, respectively. (**f**) Violin plot of distinct signature genes characteristic for each cluster. (**g**) Clinical information including histology, age, BRAF-mutation status and oncological course (progressive disease PD, or stable disease SD) (**h**) Gene Set Enrichment Analysis (GSEA) of each cluster group was performed and illustrated by bar-plots. P-values are determined GSEA and adjusted by False-Discovery Rate for multiple testing. Data is given as mean ± standard deviation; *p < 0.05, **p < 0.01, ***p < 0.001.

First, we explored the transcriptional landscape of long-term epilepsy-associated tumors by merging three independent datasets (Fig. 1a). Expression profiles were normalized and corrected from batch effects. The optimal number of clusters was determined by using a partitioning around medoids (PAM) unsupervised clustering and achieved the highest average silhouette width at four clusters (C1–C4) (Fig. 1b). Samples with negative silhouette width were removed to optimize cluster accuracy and subgroup discrimination (Supplementary Fig. 1S). The distribution of all datasets was well balanced across all clusters (Fig. 1c). Validation was performed by tSNE and consensus cluster, confirming the optimal number of 4 clusters (Fig. 1d and Supplementary Fig. 2S). All identified subgroups showed distinct signature genes and characteristic transcriptional architecture with activation of oncogenic pathways (Fig. 1e,f,h and Supplementary Fig. 2S), while their histopathological classification remained only partially preserved within the clusters (Fig. 1g). In order to exclude a potential co-clustering by low tumor content and being able to compare the specific expressional differences of the clusters to wild type, we have performed an additional expression analysis of normal brain samples (Atlas of Human Brian, http://human.brain-map.org) (Supplementary Fig. 4S).

The first cluster (“DNT-like” cluster, C1) contained only samples (n = 11), which were histologically diagnosed as DNTs (Fig. 1g). The cluster showed exclusive expression of *IRF1* (Interferon Regulatory Factor 1) and *IL10RA* (Interleukin 10 Receptor Subunit Alpha), which participate in immune regulating pathways and were independently up-regulated in this subgroup (Fig. 1e,f). Gene Set Enrichment Analysis (GSEA) confirmed these findings by identifying strong enrichment of immune response pathways, which included STAT3 activation and TGF-beta signaling (Fig. 1h). A *BRAF* mutation was not observed in this cluster and the patients from this cluster showed low recurrence rate (n = 1; p < 0.05).

The second cluster contained 18 samples with histologically diagnosed GGs or PXAs, distributed within different age groups (Fig. 1g). In contrast to all other clusters, the patients from this cluster showed a significantly higher recurrence rate of 42% (Fig. 1g). *BRAF* mutation or gain of *BRAF* was observed in 71%, which was a genetic hallmark of this subgroup (p < 0.001) (Fig. 1g). Therefore this group was designated as “BRAF-GG” (C2). *CDH18* (cadherin 18) was one of the hallmark genes of this cluster. *CDH18* is part of the cadherin family, which are known to play a crucial role in the oncogenesis by activation of EGFR and MAPK-pathways^22^. In line with these findings and the detected BRAF mutational status, we found an increased pathway signaling of the MAPK and up-stream pathways such as FGFR and EGFR (Fig. 1h and Supplementary Fig. 2S).

The third cluster (“Juvenile-GG” cluster, C3) included predominantly younger patients (n = 13, of whom 10 patients were younger than 18 years) with mainly histologically diagnosed GGs (Fig. 1g). *RAP1B* (Ras-related protein Rap-1b) and *PSMA2* (Proteosome subunit, alpha type 2) were identified as exclusively up-regulated in “Juvenile-GG”, underlying the dominant oncogenic driver of this subgroup (Fig. 1e,f). The *RAP 1* gene has been showed to play a crucial role in Notch activation and cell adhesion^23^. In line with these findings gene set enrichment analysis revealed an up-regulation of the NOTCH- and focal adhesion pathways (Fig. 1h and Supplementary Fig. 2S). One patient (7.5%) with identified BRAF mutation showed tumor recurrence during the course of the disease (p > 0.05) (Fig. 1g).

The fourth cluster contained a mixed population of histologically diagnosed DNTs and GGs samples without BRAF mutations or cases of tumor recurrence (no other specified, NOS cluster, C4). We identified up-regulated *PLXNB1* (Plexin B1) and *MBP* (myelin basic protein) (Fig. 1e,f). Here, gene set enrichment analysis showed an activation of the GABA-signaling and the nicotine-pathway (Fig. 1h) without association with any strong oncogenic driver.

### Patients’ characteristics, histopathological features and clinical implication

The clinical and molecular characteristics of all patients from the local database (n = 18) included into the transcriptional analysis (“Dataset Freiburg”) are presented on Table 1. The neuropathological examination revealed 6 GGs, 10 DNTs and 2 PXAs. All tumors were negative for *IDH*-mutation and classified as WHO grade I (except for both PXAs, which were classified as WHO grade II and III, respectively). The majority of the tumors (n = 15) were localized in the temporal lobe. The mean age at surgery was 28 years (+/−14.6) and 16 patients (88%) were seizure free after resection. Tumor recurrence was seen in four patients (one patient from “DNT-like” cluster 1 and three patients from “BRAF-GG” cluster 3) (Table 1).

**Table 1:** A summery of clinical, neuropathological and oncological characteristics of the patients with long-term epilepsy-associated tumors, who underwent transcriptional analysis.

N	Sex	Age at surgery (years)	Last available outcome (Engel)	Neuro- pathological examination	Associated FCD	Hippocampus sclerosis	WHO	CD 34	IDH mutation	MIB	Dead	Recurrence	Transcriptional signature
1	F	42	Ia	DNT	no	no	I	negative	WT	2–5%	no	no	1
2	M	28	Ia	DNT	no	no	I	positive	WT	1%	no	no	1
3	M	10	IIIa	DNT	no	no	I	positive	WT	5%	no	yes	1
4	M	48	Ia	DNT	no	no	I	negative	WT	NA	no	no	1
5	M	70	Ia	DNT	no	no	I	NA	WT	<1%	no	no	1
6	M	15	Ia	DNT	no	no	I	negative	WT	<1%	no	no	1
7	M	12	Ia	DNT	no	no	I	positive	WT	<1%	no	no	1
8	M	14	Ia	PXA	no	no	II	positive	WT	11–20%	no	yes	2
9	M	25	Ia	GG	no	no	I	negative	WT	2–5%	no	yes	2
10	F	27	Ia	GG	no	no	I	positive	WT	2–5%	no	no	2
11	F	16	Ia	GG	no	no	I	positive	WT	<1%	no	no	2
12	M	27	Ia	GG	no	no	I	positive	WT	<1%	no	no	2
13	F	20	IIIa	PXA	no	no	III	negative	WT	20%	no	yes	2
14	M	16	Ia	DNT	no	no	I	negative	WT	2–5%	no	no	4
15	M	41	Ia	GG	no	no	I	negative	WT	1%	no	no	4
16	F	33	Ia	GG	no	no	I	positive	WT	<1%	no	no	4
17	M	20	Ia	DNT	no	no	I	positive	WT	<1%	no	no	4
18	M	17	Ia	DNT	no	no	I	positive	WT	<1%	no	no	4

A summery of clinical, neuropathological and oncological characteristics of the patients with glioneuronal epilepsy-associated tumors, who underwent transcriptional analysis.

**Figure 2:**
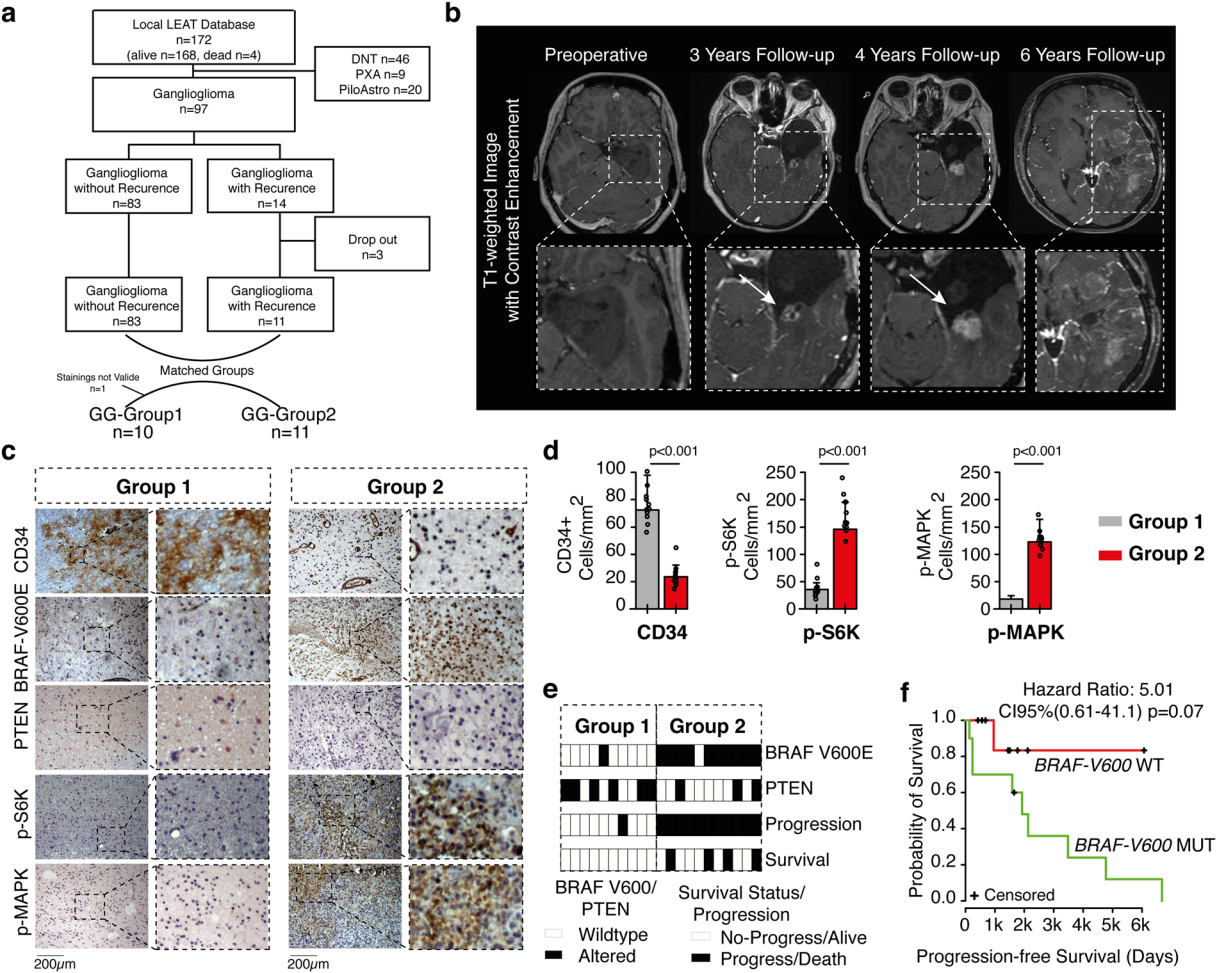
Clinical and immunochistochemical (IHC) validation of the transcriptional signature. (**a**) Selection of all patients with recurrent ganglioglioma (GG-Group 2, n = 11) from the local LEAT database. Ten patients without recurrence but matched by age, sex and tumor localization were used as a control group (GG-Group 1). (**b**) MRI example of a patient with malignant ganglioglioma. (**c**) IHC analysis of CD34, *BRAF-V600* mutation status, PTEN, p-S6K and p-MAPK in both GG-Group 1 and GG-Group 2. (**d**) The bar plots show a quantification of protein expression analysis of the CD34, p-S6K and p-MAPK signaling for both groups P-values are determined by one-way ANOVA adjusted by Benjamini-Hochberger for multiple testing. Data is given as mean ± standard deviation. (**e**) Presentation of oncological data (progression and survival) together with *BRAF-V600E* and PTEN mutation status for both groups (**f**) Kaplan-Meier-curves comparing the PFS between both groups after stratification for BRAF-V600E.

Since the transcriptional analysis revealed that the second tumor cluster (BRAF-GG) carries an increased risk for tumor recurrence (42%) we aimed at validating these results by investigating the BRAF status in patients with recurrent gangliogliomas (and without transcriptional data) from the local database. We were able to identify 14 patients with recurrent gangliogliomas (Fig. 2a). All patients have shown radiological tumor mass increase or recurrence of already resected tumors. The MRI visualization of these patients revealed local or diffuse contrast enhancement, mass effects and vast perifocal edema, which is commonly associated with high-grade glioma (Fig. 2b). Histologically proven malignant progression *(from WHO grade I to WHO grade II or III)* was observed in 6 patients. Three patients had to be excluded because of insufficient tissue quality. Clinical data of the remaining 11 patients (GG-Group2) were used to build matched pairs with patients without tumor recurrence (GG-Group1) (Fig. 2a). Both groups were evaluated for BRAF V600E mutation, p-S6K, p-MAPK, PTEN and CD34 alterations by immunohistochemistry (IHC, Fig. 2c,d). Protein targets were chosen based on our primary analysis, suggesting that activation of MAPK pathway was associated with poor oncological prognosis and early tumor progress. Since not only BRAF mutations but also other oncogenic drivers lead to activation of MAPK and AKT-mTOR pathways, our goal was to examine to what extent MAPK or AKT-mTOR pathway activation was associated with poor disease course. In GG-Group1, 9 of 10 patients (one patient was excluded because of invalid staining) showed wildtype BRAF status (Fig. 2e, Supplementary Fig. 3S). In contrast, 10 of 11 patients from the GG-Group2 (recurrent tumors) revealed BRAF-V600E mutations (Supplementary Fig. 3S) with consequent activation of the MAPK signaling and expression of S6K, implying activation of both MAPK and AKT-mTOR pathways (Fig. 2d,e). It is noteworthy that all four deceased patients from the local database died because of tumor recurrence and carried *BRAF* mutations. *BRAF* mutation showed an impact on the progression free survival (PFS) as well, sharply separating the Kaplan-Meier-curves of wildtype and mutated patients (Fig. 2f), although a statistical significance was not reached due to the low sample size.

## Discussion

Although LEATs were initially described more than 15 years ago^2^, their molecular genetic background and underlying biology still remain unknown. Differences in the clinical and oncological disease course between pathologically similar tumors suggest that the histological appearance does not reflect their biological heterogeneity^6,13^ and hint on a potential value of a further molecular-genetic characterization^24^. Recently, growing evidence has supported the hypothesis that various common oncogenic drivers are also contributing to the evolution of LEATs. Our results are consistent with these findings showing a pivotal role of EGFR/FGFR tyrosine kinase receptor and its downstream pathways RAS/RAF/MAPK and PI3K/AKT/mTOR in the pathogenesis of long-term epilepsy-associated tumors^9,25,26^. The importance of the oncological mechanisms participating in tumor pathogenesis is further emphasized by the fact that some glioneuronal tumors relapse and eventually become malignant^17^.

In this study, we demonstrate that LEATs can express distinct oncogenic drivers and are characterized by a transcriptional profile, which only partially corresponds to their histopathological appearance. The first subgroup (“DNT-like”, C1) included tumors, which were histologically diagnosed as DNTs. The oncogenic pathways of STAT3 and TGF-signaling were characteristic for this group. In line with the results of Stone and colleagues^13^, showed that BRAF mutation in DNTs was only of minor importance compared to GGs. Both the neuronal expression profile and the low recurrence rate implied the benign character of these tumors. In contrast, the second cluster (“BRAF-GG”, C2) included tumors histologically diagnosed as GGs or PXAs. BRAF alternations frequently appeared in this subgroup and were associated with poor oncological outcome and tumor recurrence in almost half of the patients. Our data suggest, that MAP/ERK, EGFR and FGFR signaling contributed to the higher recurrence rate and malignant transformation. Similar findings are observed in other malignant brain tumors as well^27^ reflecting the aggressive and proliferative nature of this subgroup. Of note, due to their similar histopathological appearance some of the PXAs could have been misdiagnosed as GGs implying the necessity for integration of DNA methylation-based classification as proposed by Capper *et al*.^24^

In order to validate these findings, we identified 10 patients with recurrent GGs from the local LEAT database. 90% of these patients revealed *BRAF* mutation along with increased MAPK signaling, confirming the transcriptional-classification findings. Additionally, we observed that PTEN expression was lost in the majority of these patients (n = 7/10, 70%) causing additional activation of the AKT-mTOR pathway marked by the increased frequency of p-S6K positive cells. The activation of both MAPK and AKT-mTOR pathways in BRAF mutated tumors was reported by other groups as well^26^. Kaplan-Meier analysis, stratifying for the BRAF mutation, revealed shorter PFS in the BRAF mutated tumors without reaching statistical significance due to the low sample number. Certainly, the reason for this is a low power resulting in a classic ß-error, which represents a limitation of the study.

Interestingly, Stone *et al*. pointed out that BRAF mutation is common for GGs, while FGFR1 mutations mainly occur in DNTs. Similar findings were investigated by other authors as well^10,28^. While we also showed that BRAF mutations are predominantly observed in histologically diagnosed ganglioglioma, we could not show an activation of the FGFR pathway in the “DNT-like” cluster. Possible explanations for this discrepancy could be differences in the examined population (adult vs. pediatric population) or in the histological classification, which may not be reliable in terms of glioneuronal pathologies. Of note, the third cluster (“Juvenile-GG”) revealed activation of the mTOR pathway without the presence of *BRAF* mutations. This implies an alternative activation of the AKT-mTOR pathway either through activating mutations or loss of function in PTEN.

The last group of LEATs included almost equally GGs and DNTs without BRAF mutation or tumors recurrences. The GSEA revealed unspecific pathway signaling including activation of neurotransmitter pathways (GABA release) and oligodengroglial gene expression, which may correspond to the *FGFR1* subgroup reported by Stone *et al*.^13^. The activation of GABA related pathways has been shown to be associated with increased epileptogenic activity by changes in chloride homeostasis, switching the GABAergic signaling from hyperpolarizing towards depolarizing^29^.

Our study has some limitations as well. Both GSEA/GSVA analysis are based on in-silico calculations, which are less robust in terms of pathway enrichments. Therefore, we have performed a further validation of the oncologically important group (“BRAF-GG”) based on immunohistochemistry (IHC). However, IHC can show some flaws particularly in cases when mutations are presented with low allele frequency highlighting by this the necessity of direct sequencing in such cases.

## Conclusion

**Figure 3:**
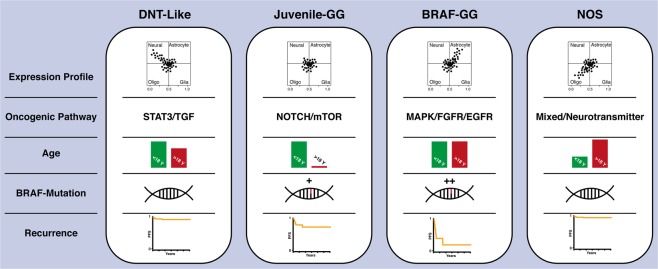
Molecular and clinical overview. Summery of expression profiles, activated oncogenic pathways, involved metabolism and progression-free survival rates of long-term epilepsy associated tumors according to their transcriptional signature.

Our transcriptional analysis map various molecular alterations involved in the development and oncological characterization of long-term epilepsy-associated tumors. Finally, we would like to discuss our findings in regard of their clinical relevance. The oncological aspects in the identified subgroups with neuronal and oligodendroglial differentiation including STAT3/TGF or neurotransmitter activation (Fig. 3) appear to be of minor importance. Consequently, surgical interventions should focus on treatment of drug-resistant epilepsy. In contrast, patients with LEATs demonstrating astrocytic differentiation, *BRAF* mutation as well as activation of MAPK/FGFR/EGFR oncogene pathways are at higher risk of tumor recurrence and malignant progression (Fig. 3). The treatment of these tumors should be performed from oncological point of view, in which gross total resection of the tumor followed by narrow clinical observation plays a pivotal role. In relapsed tumors involving functionally eloquent brain areas that restrict surgical resection, targeted therapy with BRAF inhibitors may be a feasible and promising treatment option.

## Methods and Materials

### Contact for reagent and resource sharing

Further information and requests for resources, raw data and reagents should be directed and will be fulfilled by the contact: D. H. Heiland, dieter.henrik.heiland@uniklinik-freiburg.de. Full table of all materials is given in the Supplementary Information.

### Ethical approval

In this study, we included 38 patients (18 patients for transcriptional analysis and 20 patients for immunohistochemical analysis) with long-term epilepsy-associated tumors who underwent surgery at the Department of Neurosurgery of the Medical Center, University of Freiburg. The local ethics committee of the University of Freiburg approved data evaluation, imaging procedures and experimental design (protocol 100020/09 and 5565/15). The methods were carried out in accordance with the approved guidelines. Written informed consent was obtained according to the Declaration of Helsinki.

### Imaging

MR imaging was performed on 3 T Siemens scanners (Magnetom TIM Trio, Magnetom Prisma, Siemens Medical Solutions, Erlangen, Germany) with a 32- or 40-channel head coil and an epilepsy-dedicated protocol that has been described previously. This protocol includes the following key sequences: 3D-T1-weighted MPRAGE (sagittal orientation, 160 slices, 1 × 1 × 1 mm, TI, 1100 ms; TR, 2200 ms; TE, 2.15 ms; flip angle, 12°; 7:04 min); 3D-FLAIR-SPACE (sagittal orientation, 160 slices, 1 × 1 × 1 mm, TI, 1800 ms; TR 5000 ms; TE 388 ms variable; 6:42 min); T2-STIR (coronal orientation, 40 slices, 0.4 × 0.4 × 2 mm, TI, 100 ms; TR 5300 ms; TE 24 ms; flip angle 140°, 7:59 min) and 3D-T1-weighted MPRAGE following contrast injection.

### Histology and neuropathology classification

The tissue was fixed using 4% phosphate buffered formaldehyde and paraffin-embedded through standard procedures. H&E staining was performed on 4 µm paraffin sections using standard protocol. All samples were independently reviewed according to 2016 WHO criteria by the Department of Neuropathology, Medical-Center Freiburg.

### RNA extraction and transcriptom analysis

Before RNA extraction, staining’s of the specimens were performed to confirm the presence of tumor with the lowest cut-off value 62.4% of histologically confirmed tumor tissue. RNA was extracted by All Prep Kit (Qiagen, Venlo, Netherlands) according to the manufacturer’s instructions from formalin-fixed, paraffin-embedded (FFPE) samples. RNA integrity was measured using the Agilent RNA Nano Assay Agilent Bioanalyser 2100 (http://www.home.agilent.com) according to the manufacturer’s instructions. Transcriptome analysis was performed by Clariom^TM^ D Assay (Thermo Fisher) according to the manufacturer’s instructions. Raw data were processed, normalized and controlled by R software and the Affymetrix R-package. Different expression analysis and statistical testing (pairwise t-test) were performed by limma R-package. (Data available in GEO: GSE108013).

### Data post-processing, integration of GEO database

Two datasets of LEATs from the GEO database were integrated into our analysis (GSE60898 and GSE94349). A total number of 47 samples with the histology of DNTs, GGs or PXAs were taken into consideration. In a first step, all data were aligned to their Entrez Gene^30^ in according to common practice. Next, we removed batch effects by ComBat algorithm^31^, normalized and scaled the data. Finally, all datasets were merged and contained a total of 65 patients.

### Semi-Supervised cluster analysis

First, to assess clustering robustness, we used two different algorithms to validate the optimal number of clusters. All patients were analyzed by the ConsensusClusterPlus^32^. The optimal number of clusters was defined by the highest area under the CDF (Consensus Cumulative Distribution Function) curve. In a second analysis, a ‘partitioning around medoids (PAM) cluster analysis was performed on the 1000 most variable genes to identify robust metabolic clusters (‘PAM’ function of the ‘cluster’ package in R^33^). To determine the optimal number of clusters, mean silhouette widths were computed (clustering repeated from k = 2 to k = 10) and confirmed an optimal number of four clusters, which was chosen for downstream analysis. In order to identify the “core” patients of each cluster, we further excluded samples with negative silhouette widths, retaining 53 patients.

### Differential gene expression of long-term epilepsy-associated tumors transcriptional subgroups

We aimed to identify the “core” genes of each cluster by a large-scale regression model (Supplementary Table 1S). The model aligns all genes based on their affiliation-likelihood within all four subgroups. The resulted score and its negative logarithm of the FDR was visualized in a scatterplot, in which different axis presented an individual subgroup. Based on the regression model, subclass scores were used to identify subgroup specific pathway activation.

### Gene set enrichment analysis

A permutation-based pre-ranked GSEA was applied to each module to verify its biological functions and pathways^34^. We compute genes that survive the thresholding, from the nearest shrunken centroid classifier. The average rank of the gene in the cross-validation folds for genes surviving at the given threshold was used for GSEA as described in the AutoPipe package. The predefined gene sets of the Molecular Signature Database v5.1were taken^35^. Enrichment score was calculated by the rank order of gene computed by above described classifier score of each subgroup. For significant enrichment, p-values were adjusted by FDR. Gene Set Variation Analysis (GSVA) was performed with the GSVA package implemented in R-software^36^. The analysis based on a non-parametric unsupervised approach, which transformed a classic gene matrix (gene-by-sample) into a gene set by sample matrix resulted in an enrichment score for each sample and pathway.

### Immunostaining

Tissue samples were fixed using 4% phosphate buffered formaldehyde and paraffin-embedded according to standard procedures. H&E staining was performed on 4 µm paraffin sections using standard protocols. Immunohistochemistry was performed on 3 µm paraffin-embedded tissue sections after deparaffinization and heat-induced epitope retrieval in citrate buffer by using the SignalStain Kit by Cell Signaling according to the manufacturer’s instructions. As primary antibody, anti-BRAF-V600 antibody (1:500, SAB5600047 SIGMA) (Supplementary Fig. 3S), anti-S6K (1:250 Anti-P70 S6 Kinase beta antibody, ab70963), anti-CD34 (1:600, Anti-CD34 antibody [EP373Y], ab81289), anti-p38 MAPK (1:1000, Anti-p38 antibody, ab197348) and anti-PTEN (1:1000, Anti-PTEN antibody, ab31392) was applied to the tissue and incubated overnight at 4 °C. The next day, after application of SignalStain® Boost IHC Reagent followed by SignalStain® DAB, counterstaining with Meyer’s haemalaun solution was performed. The samples were then mounted and analyzed with an Olympus microscope. Positive cells were counted (by ImageJ) in 6 high-fields (40x magnification) per slide and compared to the total number of cells in each field. From this data, the mean percentage of positive cells was calculated.

### Statistical analysis

Distribution and variances of all data was tested by Shapiro-Wilk test (p < 0.05) to confirm normality. We tested the difference between all groups or cluster groups by Wilcoxon signed-rank test (unpaired) and determined a 5% alpha-level. All statistical analysis was performed with R-software. The Kaplan-Meier method was used to provide median point estimates and time-specific rates. The Hazard-Ratio (HR) was calculated using Cox-Regression tests.

## Supplementary information

Supplementary Information 
