# Supplementary material for: Long-term epilepsy-associated tumors: transcriptional signatures reflect clinical course
Source: Sci Rep. 2020 Jan 9;10:96. doi: 10.1038/s41598-019-56146-y (PMC6952384; doi:10.1038/s41598-019-56146-y)
Supplement: Supplementary file 1 — Supplementary Information [file 41598_2019_56146_MOESM1_ESM.zip › Supplementary file/Legends for supplementary figures and tables.docx]

**Legends for supplementary figures and tables**

**Supplementary Fig. 1S Cluster analysis of full dataset**

a) Data of the full dataset without outlier excluding. Bar-plot of the silhouette widths of each patient based on the “PAM” cluster integrated in the AutoPipe-package (CRAN). The optimal number of clusters was computed by “PAM” clustering and visualized by the mean silhouette widths. b) Sample distribution among all integrated datasets c) A heatmap with distinct up- and downregulated genes of each cluster group (C1-C4). *Red* indicates up-regulated gene expression, *blue* down-regulated genes, respectively.

**Supplementary Fig. 2S Consensus cluster and geneset variation analysis**

A) Consensus cluster of k=4 confirmed the optimal number of 4 cluster groups. A heatmap of patient-patient correlation matrix is displayed. Blue color marked high correlation, white color low correlation respectively. B) Geneset variation analysis (GSVA) was performed in order to validate the GSEA. The given heatmap shows pathway activity of described pathways (on right side) with their multiple genesets (C2, Molecular Signature Database).

**Supplementary Fig. 3S Immunohistochemistry analysis of BRAF mutation in both GG-Group1 and GG-Group2** Each patient is shown on a single row.

**Supplementary Fig. 4S A heatmap with distinct up- and downregulated genes of each cluster group (C1-C4, and normal brain)**. *Red* indicates up-regulated gene expression, *blue* down-regulated genes, respectively**.** Normal brain control samples are used from the human brain atlas (http://human.brain-map.org/).

**Supplementary Table 1S Signature genes of each cluster**
