# Supplementary figures and images for: Long-term epilepsy-associated tumors: transcriptional signatures reflect clinical course
Source: Sci Rep. 2020 Jan 9;10:96. doi: 10.1038/s41598-019-56146-y (PMC6952384; doi:10.1038/s41598-019-56146-y)

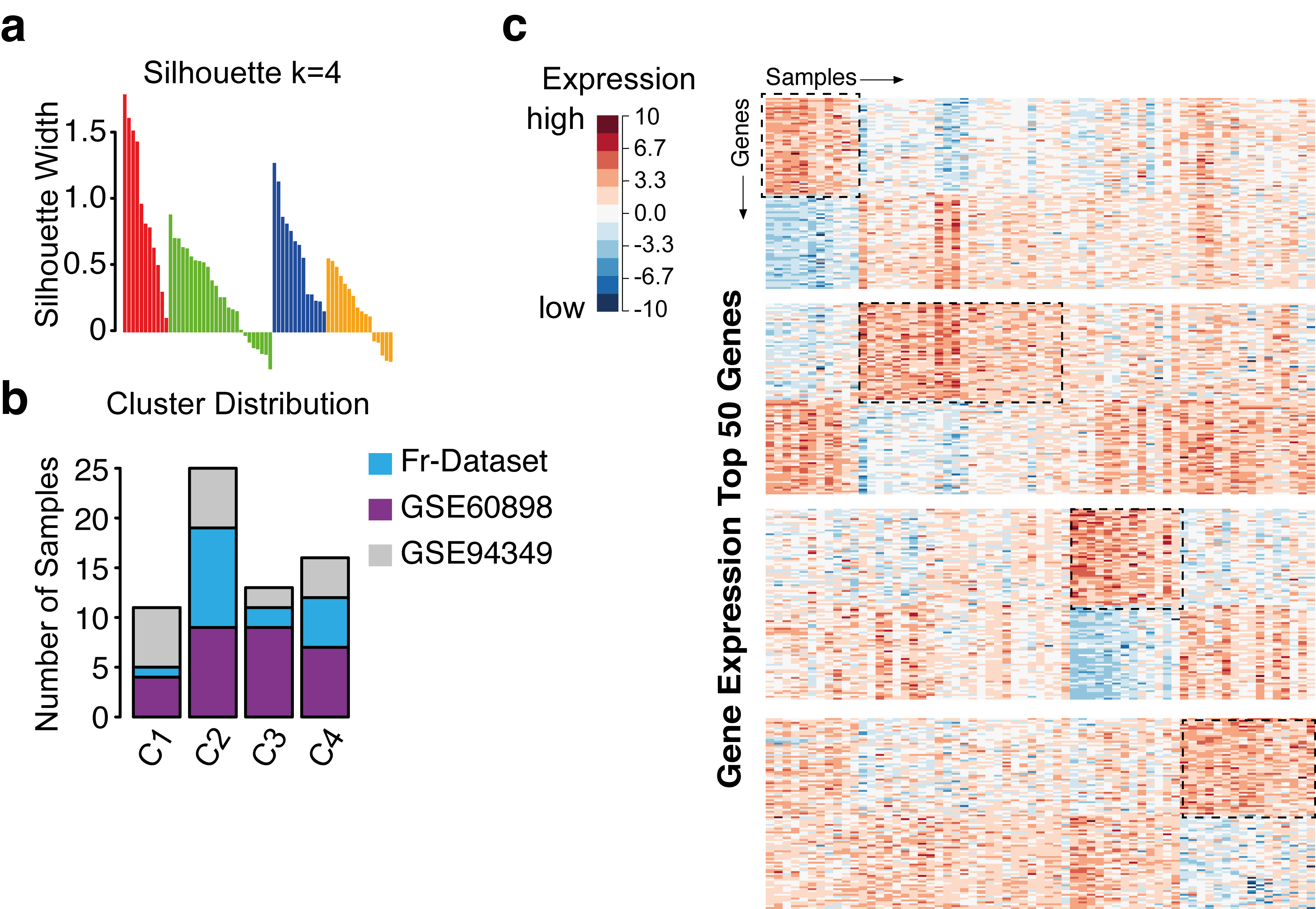

Supplement: Supplementary file 1 — Supplementary Information [file 41598_2019_56146_MOESM1_ESM.zip › Supplementary file/Supplementary Fig.1S.png]

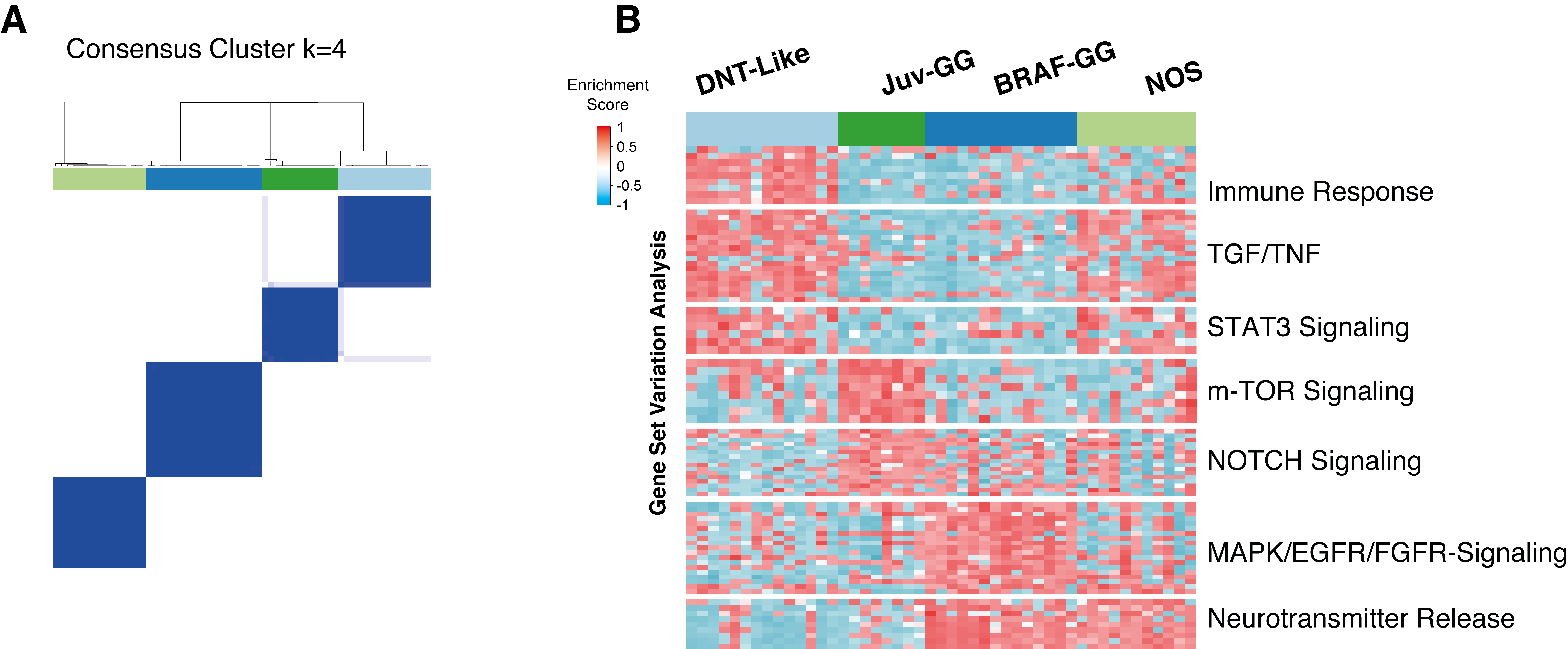

Supplement: Supplementary file 1 — Supplementary Information [file 41598_2019_56146_MOESM1_ESM.zip › Supplementary file/Supplementary Fig.2S.png]

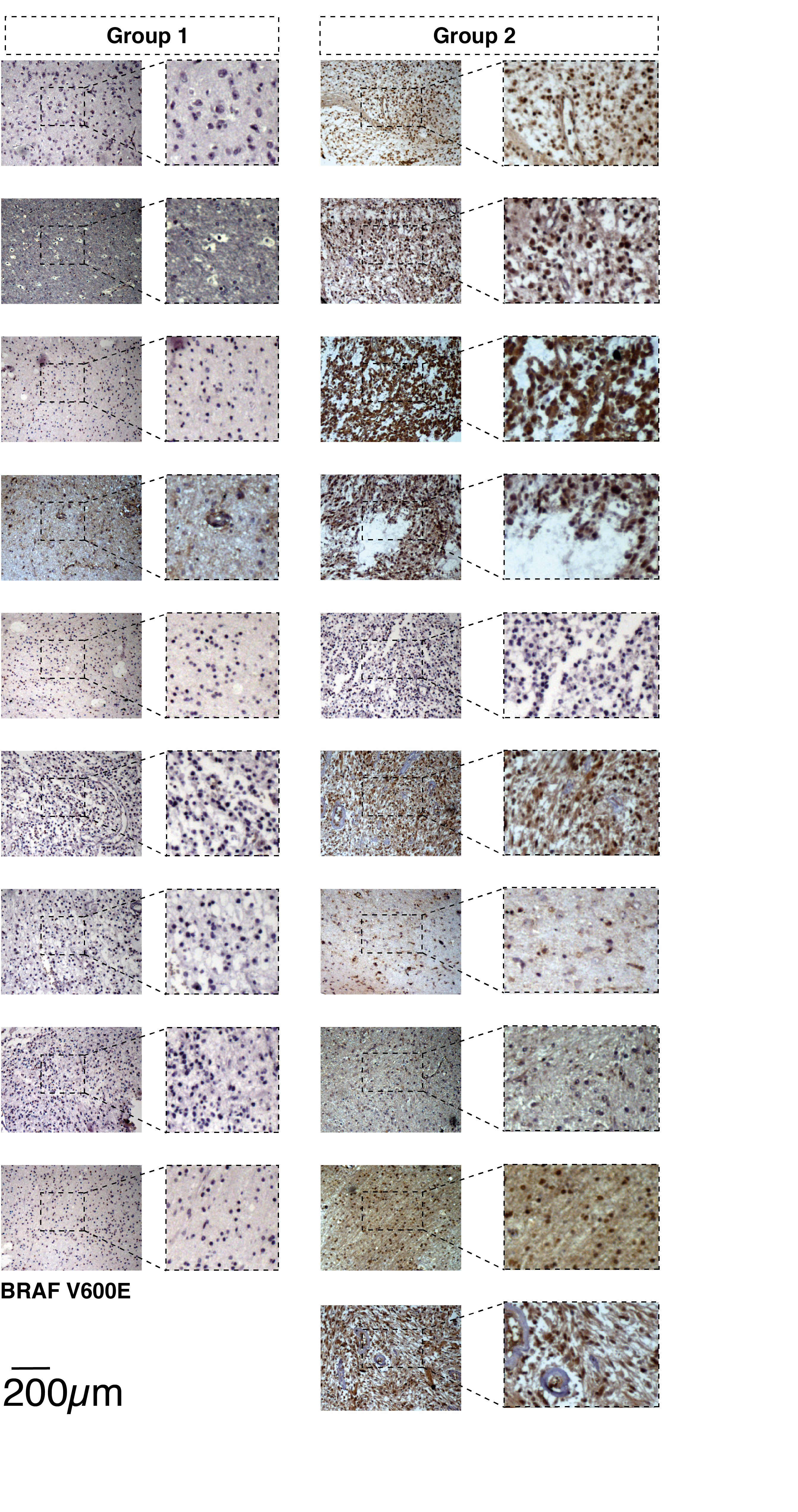

Supplement: Supplementary file 1 — Supplementary Information [file 41598_2019_56146_MOESM1_ESM.zip › Supplementary file/Supplementary Fig.3S.png]

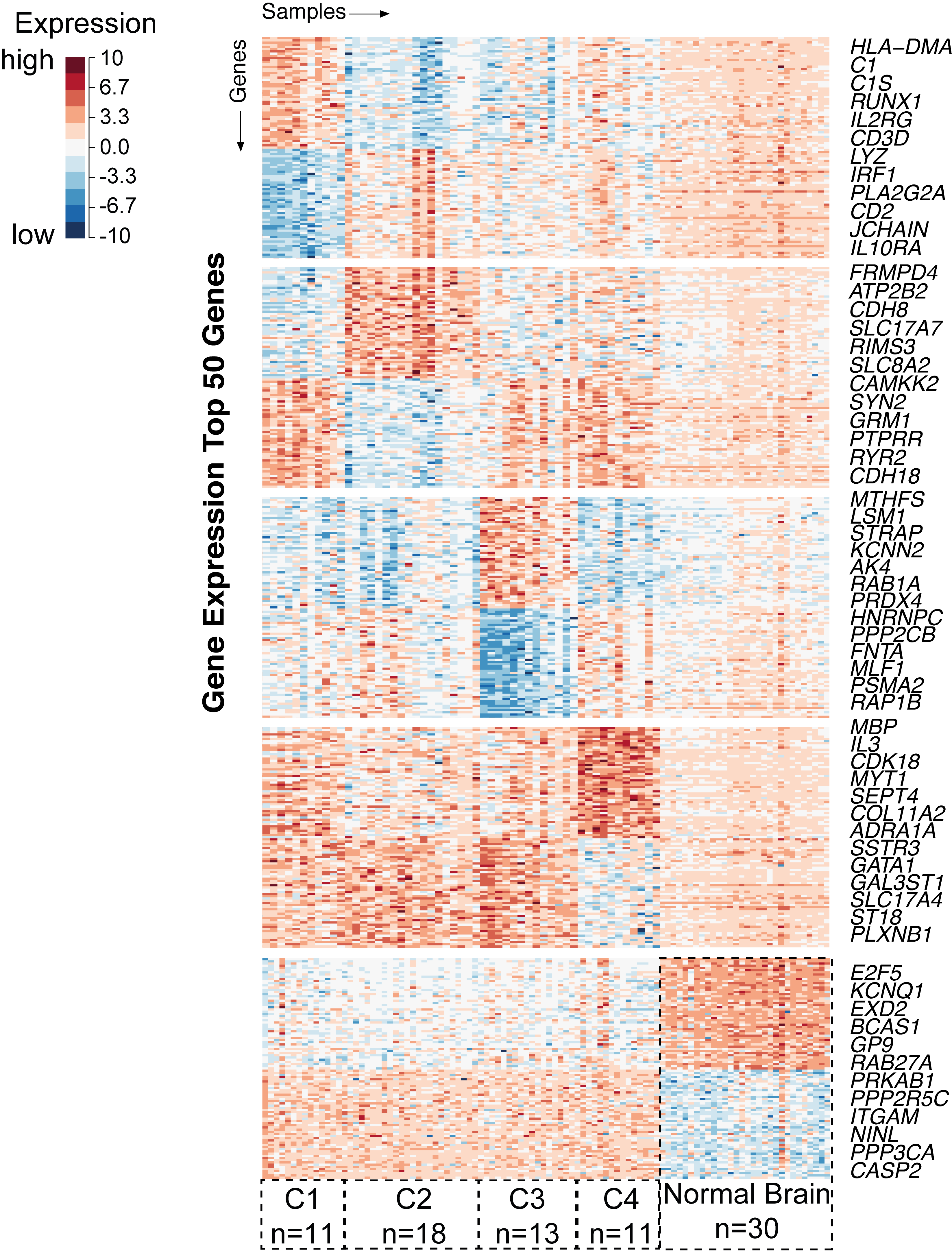

Supplement: Supplementary file 1 — Supplementary Information [file 41598_2019_56146_MOESM1_ESM.zip › Supplementary file/Supplementary Fig.4S.png]
